# Editorial: Early signaling in the rhizobium-legume symbiosis

**DOI:** 10.3389/fpls.2022.1056830

**Published:** 2022-10-19

**Authors:** María J. Soto, Christian Staehelin, Benjamin Gourion, Luis Cárdenas, José M. Vinardell

**Affiliations:** ^1^ Genetics of Phytobacterial Infections, Estación Experimental del Zaidín, Department of Biotechnology and Environmental Protection, CSIC, Granada, Spain; ^2^ State Key Laboratory of Biocontrol and Guangdong Key Laboratory of Plant Resources, School of Life Sciences, Sun Yat-sen University, Guangzhou, China; ^3^ LIPME, INRAE, CNRS, Université de Toulouse, Castanet-Tolosan, France; ^4^ Instituto de Biotecnología, Universidad Nacional Autónoma de México, Morelos, Mexico; ^5^ Department of Microbiology, University of Sevilla, Sevilla, Spain

**Keywords:** rhizosphere, chemotaxis, plant nodulation genes, exopolysaccharides, effectors, volatiles

The rhizobium-legume symbiosis is a beneficial plant-bacteria association between soil bacteria collectively known as rhizobia and leguminous plants (Poole et al., 2018). To establish symbiosis, the rhizobia invade roots of host plants and trigger the formation of a new organ, the nodule. The rhizobia differentiate then into bacteroids and reduce atmospheric nitrogen into ammonia that will be used by the plant. The formation of nitrogen-fixing nodules is a complex process in which bacterial infection needs to be co-ordinated with the nodule organogenesis program. This is achieved through a continuous molecular dialogue between the host plant and rhizobia, which leads to a high degree of specificity in the interaction with even strain- and host cultivar-dependent effects (Cangioli et al., 2022). Much of that specificity is conferred by an exchange of signals that take place in the rhizosphere during the early stages of the association (Roy et al., 2020). The 10 articles hosted in the Research Topic “Early signaling in the rhizobium-legume symbiosis”, increase our knowledge on the molecular bases underlying the mutual recognition of plants and rhizobia during early stages of the interaction, i.e., before the onset of nitrogen fixation.

The initiation of the rhizobium-legume symbiosis requires localization of the bacteria to potential infection sites on host roots. In this process, rhizobial chemotaxis and motility play a relevant role. The reviews by Aroney et al. and Compton and Scharf revised some important concepts related to motility and chemotaxis and the preferred attractants of several model rhizobia. Aroney et al. highlighted the importance of understanding chemotaxis and motility in legume symbionts, especially under the environmental conditions found in the field. This, together with the identification of different compounds sensed through the chemotaxis systems in each rhizobial species, might predict symbiotic performance based on specific environmental conditions and root exudate composition, making the selection of elite inoculants more accurate.

The role of (iso)flavonoids as chemoattractants in rhizobia is not clear. However, they are crucial in the initiation of the symbiosis by triggering production of specific lipochitooligosaccharidic Nod-factors (NFs) in the rhizobial partner. Perception of NFs by specific plant receptors activates the symbiosis signaling pathway required for rhizobial infection and nodule formation. Three studies of the article collection provide insights on early plant symbiotic genes. Solovev et al. have addressed the difference in symbiotic specificity between Afghanistan and European pea landraces, in which the former can interact only with *Rhizobium leguminosarum* bv. *viciae* strains that have the *nodX* gene and produce NFs with an additional acetyl group at the reducing end while European pea lines can also be nodulated by strains lacking *nodX*. By using molecular modelling of putative receptor heterodimers with different NFs, the authors identified the *LykX* receptor gene as a possible determinant of pea specificity toward *nodX*-containing strains. Kovacs et al. have investigated the protein complex formed by NSP1 and NSP2, two transcriptional regulators of *Medicago truncatula* required for the activation of several early nodulation genes. Specifically, the authors addressed amino acid polymorphisms in the VHIID motif of NSP2. Characterization of a mutant allele unveiled the importance of a conserved Asp residue in NSP2, which is essential for the formation of a functional NSP1-NSP2 signaling module. Root hair deformation and infection thread formation require an important cell reorganization during which Rho GTPases called ROPs (for Rho of plants) play key roles. García-Soto et al. reported on ROP3, a novel Rho-GTPase of *Lotus japonicus*. The study shows that, in response to rhizobial inoculation, *rop3* mutant plants exhibit altered root hair deformation and expression of nodulation-related genes, and form fewer nodules than wild-type plants, indicating that ROP3 is a positive regulator of rhizobial infection thread formation.

In addition to NFs, rhizobial surface polysaccharides and effector proteins delivered by specialised secretion systems play important roles in the success of the interaction, very often in a host-specific manner (Acosta-Jurado et al., 2021; Jiménez-Guerrero et al., 2022). A good example of that has been provided by Castellani et al. who analysed the symbiotic relevance of the exopolysaccharide (EPS) of *Rhizobium favelukesii* LPU83, a strain which like *Sinorhizobium meliloti* is able to nodulate alfalfa. Interestingly, although both strains produce the same EPS, this polysaccharide is essential for a successful interaction of alfalfa with *S. meliloti*, but not with *R. favelukesii*. The production of symbiotically important EPSs is under the control of different regulatory systems, which are also relevant for the symbiosis. In *S. meliloti*, one of them is the ExoS/ChvI two-component regulatory system, which is normally activated in rhizobial cells during the transition from a free-living to a symbiotic life style. Mutations that increase or decrease activity of the system lead to symbiotic defects. Geiger et al. demonstrated that the lack of the phospholipid phosphatidylcholine (PC) in the sinorhizobial membrane leads to permanent activation of ExoS/ChvI, which might explain the inability of a PC-deficient mutant to form nodules on alfalfa roots.

The type 3 secretion system (T3SS) is the main protein secretion apparatus known to have an impact in the symbiosis. Ratu et al. showed that the T3SS effector protein Bel2-5 of *Bradyrhizobium elkanii* USDA61 possesses multiple domains that might interact and modify host targets and provoke opposite roles on symbiosis with soybean depending on the plant genotype: Bel2-5 promotes NF-independent symbiosis with a *nfr1* soybean mutant but blocks nodulation with soybean plants carrying the *Rj4* allele. Besides the most relevant T3SS, recent studies have shown the participation of additional rhizobial protein secretion systems in the symbiotic association. Hug et al. studied type 6 secretion systems (T6SSs) of *Paraburkholderia phymatum* STM815. This strain is able to nodulate a broad range of legumes and its T6SS-b appears to play a role in bacterial motility. T6SS-b gene expression was found to be activated in the presence of citrate and germinated seeds of host legumes.

The inter-kingdom signaling between rhizobia and legumes is complex and additional players, which might not be essential for nodulation but could contribute to fine-tune some aspects of the symbiosis, could be added to the chemical dialogue. Based on the recognized role played by microbial volatiles in inter-kingdom signaling with plants (Weisskopf et al., 2021), Soto et al. have raised the possibility that rhizobial volatiles might play a signaling role in their interaction with legumes, whose precise role in symbiosis requires further investigation.

## Author contributions

MS wrote the article with contributions from JV, CS, BG, and LC. All authors contributed to the article and approved the submitted version.

## Funding

MS was supported by grants PGC2018-096477-B-I00 and PID2021-123540NB-I00 funded by MCIN/AEI/10.13039/501100011033 and by “ERDF A way of making Europe” and by grant P20_00225 funded by the Junta de Andalucía PAIDI/FEDER/EU. CS was supported by grants 31670241 and 32161133026 funded by the National Natural Science Foundation of China. JV was supported by the Spanish Ministry of Science and Innovation, grant number PID2019-107634RB-I00, and by Junta de Andalucía PAIDI/FEDER/EU, grant number P20_00185.

## Acknowledgments

The editors would like to thank all the authors and expert reviewers who have participated in the preparation and evaluation of manuscripts hosted in this Research Topic.

## Conflict of interest

The authors declare that the research was conducted in the absence of any commercial or financial relationships that could be construed as a potential conflict of interest.

## Publisher’s note

All claims expressed in this article are solely those of the authors and do not necessarily represent those of their affiliated organizations, or those of the publisher, the editors and the reviewers. Any product that may be evaluated in this article, or claim that may be made by its manufacturer, is not guaranteed or endorsed by the publisher.
